# Effect of *spd*C gene expression on virulence and antibiotic resistance in clinical *Staphylococcus aureus* isolates

**DOI:** 10.1007/s10123-022-00249-6

**Published:** 2022-05-24

**Authors:** Mayada E. Bakr, Mona T. Kashef, Alaa El-Dien M. S. Hosny, Mohammed A. Ramadan

**Affiliations:** 1 Department of Microbiology and Immunology, Faculty of Pharmacy, Modern University for Technology and Information, Cairo, Egypt; 2 Department of Microbiology and Immunology, Faculty of Pharmacy, Cairo University, Cairo, 11562 Egypt

**Keywords:** Antimicrobial resistance, Biofilm, Hemolysin, Protease, Surface protein display C (SpdC), Virulence

## Abstract

Surface protein display C (SpdC) protein was described as a novel virulence factor of *Staphylococcus aureus* that affects biofilm formation and pathogenesis and favors resistance to antimicrobials targeting cell wall. We evaluated the possible correlation between *spd*C gene expression level and virulence as well as antibiotic resistance phenotypes in *S. aureus* clinical isolates. The antimicrobial susceptibility of *S. aureus* clinical isolates (*n* = 100) was determined by the disk diffusion method. Vancomycin susceptibility was determined by the broth microdilution method. The level of the extracellular proteases and delta-hemolysin was evaluated by measuring the proteolysis and hemolysis zone diameters in skim milk and blood agar plates, respectively. Biofilm formation was assayed using the 96-well microtiter plate method. Most of the isolates (81%) were multidrug-resistant and about half of the isolates (49%) were methicillin-resistant *S. aureus*. Hemolysin, protease, and biofilm production were detectable in 79%, 71%, and 96% of the isolates. No significant correlation was detectable between the level of *spd*C gene expression and the activity of tested virulence factors or the antimicrobial resistance phenotype. Therefore, the role of SpdC protein as a virulence regulator in *S. aureus* needs further evaluation together with the determination of the predominant regulators for each virulence factor.

**Supplementary Information:**

The online version contains supplementary material available at 10.1007/s10123-022-00249-6.

## Introduction

*Staphylococcus aureus* is a highly virulent human pathogen implicated in many diseases; it causes both nosocomial and community-acquired infections (Uhlemann et al. 2014) and can result in bacteremia with significant rates of morbidity and mortality (van Hal et al. 2012). *S. aureus* infections range from localized skin infections such as folliculitis, furuncles, carbuncles, and impetigo to deep infections that spread systemically. Systemic infections involve bacteremia (with and without endocarditis); bones, joints, deep organs, and tissue infections; scalded skin syndrome in neonates; toxic shock syndrome; and food poisoning (Tong et al. 2015). According to Centers for Disease Control and Prevention (CDC), people with chronic conditions and hospitalized patients are at high risk of *S. aureus* infections (CDC 2011).

*S. aureus* is increasingly becoming resistant to several antimicrobial agents which is a challenging problem in clinical practice (Naimi et al. 2017). Methicillin-resistant *S. aureus* (MRSA) has been listed by the World Health Organization as one of the high priority pathogens for which new antibiotics are urgently needed (World Health Organization 2017). People infected with MRSA are 64% more likely to die than those infected with methicillin susceptible *S. aureus* (MSSA)(World Health Organization 2020). MRSA has also been listed by CDC as a serious threat (CDC 2019).

Vancomycin is the drug of choice for treatment of MRSA infection. The emergence of vancomycin-resistant *S. aureus* has posed additional challenge in the treatment of *S. aureus* infections, where other antimicrobial agents have been approved for the treatment of MRSA infections including daptomycin, linezolid, tedizolid, oritavancin, dalbavancin, ceftaroline, and ceftobiprole (Boswihi and Udo 2018).

Many virulence determinants were identified in *S. aureus*. Surface proteins such as adhesins, clumping factors, iron-regulated surface determinant (IsdA), fibrinogen-binding proteins, and fibronectin-binding proteins are responsible for attachment to host cells and tissue colonization. Invasins like leukocidin kinase and hyaluronidase promote bacterial spread in tissues. Other virulence determinants include surface factors (capsule and protein A) that inhibit phagocytic engulfment; and molecules that enhance their survival in phagocytes (carotenoids and catalase production). In addition, *S. aureus* produces several immunological disguises (protein A and coagulase), membrane-damaging toxins (hemolysins, leukotoxin, and leukocidin) that lyse eukaryotic cell membranes, and exotoxins that damage host tissues and provoke symptoms of disease (Reddy et al. 2017). Different types of sortase enzyme, that are considered virulence determinants through anchoring different proteins into the bacterial cell wall, were reported in *S. aureus*; these proteins have many functions in evasion of host defense and adhesion to target tissues and may function in biofilm formation (Nitulescu et al. 2021).

*S. aureus* also encodes about 16 two-component systems (TCS), representing an important regulatory mechanism that affects survival and virulence under infection conditions. The TCS encodes a histidine kinase that is usually membrane bound and senses different signals causing its autophosphorylation and a response regulator that is subsequently accepting the phosphoryl group from the histidine kinase and affects the activity of many chromosomal operons (Bleul et al. 2022).

Recently, Poupel and colleagues have described *spd*C gene (also known as *Lyr*A) as a global *S. aureus* virulence factor (Poupel et al. 2018). It encodes a transmembrane protein with ABI domain, an element first described in lactococci for its role in phage exclusion function (Frankel et al. 2010). Expression of *spd*C gene affects biofilm formation and pathogenesis and is positively regulated by the WalKR system, one of the *S. aureus* TCS (Dubrac et al. 2008). SpdC protein interacts with the WalK histidine kinase to inhibit the activity of the WalKR TCS and regulates nine other histidine kinases of *S. aureus*, suggesting its role as a global regulator (Poupel et al. 2018). *spd*C gene was found to be encoded in all sequenced staphylococci strains (Gründling et al. 2006); however, deletion of *spd*C gene was reported in a vancomycin intermediate–resistant strain (Yamaguchi et al. 2019). It was hypothesized that deletion of *spd*C gene may impact vancomycin resistance through its effect on WalKR regulon.

Poupel and colleagues reported one hundred genes whose expression is regulated by SpdC protein. The *spd*C mutant, prepared by deletion of *spd*C gene in the parent *S. aureus* strain HG001, displayed altered resistance towards compounds targeting the cell wall; it was highly sensitive to oxacillin and tunicamycin, but not to fosfomycin, which inhibits the first step of cell wall biosynthesis. Additionally, the *spd*C mutant had a diminished biofilm formation and reduced virulence (Poupel et al. 2018). Attenuation of *S. aureus* virulence can be utilized as a strategy for treatment of resistant infections (Mahdally et al. 2021).

In this study, the possible correlation between *spd*C gene expression level and the virulence as well as the resistance to different antimicrobials, in *S. aureus* clinical isolates, was evaluated.

## Materials and methods

### Microbial strains and culture conditions

*S. aureus* standard strain ATCC 25923 was used as a reference strain. *S. aureus* clinical isolates (*n* = 100) were obtained from Ain Shams Specialized Hospital (H1) and El-Demerdash Specialized Hospital (H2), between November 2018 and August 2019. The source of each isolate is indicated in Supplementary Table 1. The identification of isolates as *S. aureus* was confirmed phenotypically by Gram staining, DNase test, positive coagulase test, and mannitol fermentation (yellow colonies on mannitol salt agar media) (Procop et al. 2017) and genotypically by detection of thermonuclease (*nuc*) gene using polymerase chain reaction (PCR). The isolates were stored in tryptone soya broth (TSB) containing 25% glycerol at − 80 °C. Unless otherwise described, they were isolated on brain–heart infusion agar plates and incubated at 37 °C prior to use.

### Antimicrobial susceptibility testing

Antimicrobial susceptibility testing was carried out using the Kirby-Bauer disk diffusion method, according to Clinical and Laboratory Standards Institute’s (CLSI) guidelines (CLSI 2012a). *S. aureus* standard strain ATCC 25923 was used as a control strain. Fresh bacterial colonies were suspended in saline to reach an optical density (OD) equivalent to that of 0.5 McFarland turbidity standard (about 1 to 2 × 10^8^ CFU/mL) and then spread onto the surface of agar plates using sterile swabs. Antibiotic disks were placed onto the surface of the agar plates; the tested antibiotics were penicillin (10 µg), gentamicin (10 μg), erythromycin (15 µg), ciprofloxacin (5 μg), tetracycline (30 µg), cefoxitin (30 μg), sulfamethoxazole/trimethoprim (1.25/23.75 μg), clindamycin (2 µg), linezolid (30 µg), and chloramphenicol (30 µg). The selection of antimicrobial agents was based on CLSI-suggested antimicrobials for routine testing of *S. aureus* (CLSI 2017) and the suggested agents for defining multidrug resistance (MDR) in *S. aureus* according to the definition of Magiorakos et al. (2012). All disks were obtained from Bioanalyse, Turkey. The plates were incubated at 37 °C for 24 h. The diameters of the inhibition zones were measured and the results were interpreted according to CLSI guidelines (CLSI 2017). The breakpoints used for defining the susceptibility pattern of tested isolates are given in Supplementary Table 2.

The antimicrobial susceptibility of the isolates to vancomycin was determined by the broth microdilution method according to CLSI guidelines (CLSI 2012b). *S. aureus* ATCC 29213 was used as a quality control strain (CLSI 2017). Vancomycin (Mylan, Ireland) was tested in concentration range from 0.5 to 64 μg/mL. The 96-well microtiter plates were incubated at 37 °C for 24 h. The minimum inhibitory concentration (MIC) was defined as the least concentration that completely inhibited the growth of the microorganisms. *S. aureus* isolates with vancomycin MICs ≤ 2 μg/mL were considered susceptible. Vancomycin intermediate *S. aureus* had MICs of 4–8 μg/mL, while vancomycin-resistant *S. aureus* had MIC of ≥ 16 μg/mL (CLSI 2017).

MRSA strains were defined as *S. aureus* strains that were resistant to methicillin; cefoxitin was used as methicillin surrogate in MRSA testing (CLSI 2017). *S. aureus* isolates were categorized as MDR if they were resistant to at least one agent in three or more antimicrobial categories or were MRSA, according to the definition of Magiorakos et al. (2012).

### Determination of virulence in tested *S. aureus* isolates

#### Delta-hemolysin activity

Overnight cultures of the tested isolates in Muller Hinton broth (MHB; HiMedia, India) were diluted to reach an OD equivalent to one at 600 nm. The diluted culture (50 µL) was added into holes (5 mm in diameter) made in 7% sheep-blood agar plates. The plates were incubated overnight at 37 °C followed by storage overnight at 4 °C. *S. aureus* standard strain ATCC 25923 was used as a positive control (Zhang et al. 2016), while uninoculated MHB was used as negative control. Diameters of the hemolysis zones, in blood agar, were measured as indicative of hemolysin activity (Quiblier et al. 2011). Isolates were given arbitrary scores according to zone diameters as follows: score = 0 if hemolysis zone diameter ≤ 5 mm, score = 1 (weak hemolytic activity) if hemolysis zone diameter > 5 mm and ≤ 10 mm, score = 2 (moderate hemolytic activity) if hemolysis zone diameter > 10 mm and ≤ 20 mm, score = 3 (high hemolytic activity) if hemolysis zone diameter > 20 mm.

#### Protease activity

The assay of protease activity was performed by applying the same method described under hemolysin activity except that skim milk agar plates (Conda, Spain) were used instead of blood agar plates. Plates were incubated at 37 °C for 24 h. *S. aureus* ATCC 25923 was used as a positive control (Kaur et al. 2017), while uninoculated MHB was used as negative control. Diameters of the proteolysis zones, in skim milk agar, were measured as indicative of protease activity (Quiblier et al. 2011). Isolates were given arbitrary scores according to zone diameters as follows: score = 0 if proteolysis zone diameter ≤ 5 mm, score = 1 (weak proteolytic activity) if proteolysis zone diameter ˃ 5 mm and ≤ 10 mm, score = 2 (moderate proteolytic activity) if proteolysis zone diameter ˃ 10 mm and ≤ 20 mm, score = 3 (high proteolytic activity) if proteolysis zone diameter ˃ 20 mm.

#### Assay of biofilm production

Biofilm production was determined according to the method of Christensen et al. (1985). Briefly, overnight cultures of the tested isolates in TSB (HiMedia, India) were diluted 1:100 with the same media. Diluted samples were added in the wells of a sterile 96-polystyrene microtiter plate. Each strain was tested in triplicate. Wells with uninoculated TSB were used as a negative control. *S. aureus* ATCC 25923 was used as a positive control (Kashef et al. 2020). The plates were incubated at 37 °C for 24 h and the OD of the cultures was measured at 600 nm. The culture was then discarded, and the wells were washed with phosphate buffered saline three times. The plates were left to dry followed by addition of 200 µL of 1% crystal violet for 30 min. Excess dye was removed and the plates were washed with distilled water three times and left to dry. To measure the OD of the stained biofilms, 200 µL of 95% ethyl alcohol was added to each well, and the plate was left for 15 min; then, 125 µL of each solution was transferred to a well in a new plate. The absorbance was measured at 570 nm using enzyme-linked immunosorbent assay plate reader (Torlak et al. 2017). The cutoff OD value (Odc) was determined as the mean plus three times the standard deviations of the negative control. The OD of stained biofilm formed by each sample was normalized to the OD of the culture measured at 600 nm. If normalized sample optical density (Ods) ≤ Odc, then it is non-biofilm producer (score = 0). If Odc < Ods ≤ 2 × Odc, the sample is a weak biofilm producer (score = 1). If 2 × Odc < Ods ≤ 4 × Odc, the sample is a moderate biofilm producer (score = 2) and the sample is considered strong biofilm producer if 4 × Odc < Ods (score = 3) (Mesrati et al. 2018).

### Detection of *spd*C gene and its expression level

#### Detection of *spd*C gene

The detectability of *spd*C gene in the collected isolates was determined using PCR. To ensure conservation of *spd*C gene in *S. aureus* isolates, 78 sequences of *spd*C gene were randomly selected from the *spd*C gene sequences available at the GenBank and aligned using Sequence Alignment Tool of Clustal Omega software (https://www.ebi.ac.uk/Tools/msa/clustalo/).

Oligonucleotides were designed using the Primer Quest Tool of Integrated DNA Technologies (IDTDNA, Coralville, USA). Primer-Blast (National Center for Biotechnology Information, https://www.ncbi.nlm.nih.gov/) was used to test the specificity of the designed primers. The sequences of the used primers were as follows: *Spd*C*_Forward*: GCTTCAATGACATTTGGCCTTA, and *Spd*C*_Reverse:* CTGCAACGATTGCTGTTGAAATG. Genomic DNA was extracted by the boiling method (Sambrook and Russell 2001). The PCR reaction mixture (25 µL) contained 5 µL of bacterial lysate, 5 µL of 5 × Green GoTaq Reaction Buffer, 1 mM MgCl_2_, 0.2 mM dNTP mix, 10 pmol of each primer, and 0.625 U GoTaq DNA Polymerase. All PCR reagents were from Promega (USA). The reaction was carried out in a thermal cycler using initial denaturation at 94 °C for 3 min, 30 cycles of denaturation at 94 °C for 30 s, annealing at 60 °C for 30 s and extension at 72 °C for 1 min, and a final extension step at 72 °C for 10 min. Amplicons with the expected size (≈100 bp) were visualized by electrophoresis on 1.5% agarose gel stained with ethidium bromide.

To further confirm the specificity of the PCR product, the product from the standard *S. aureus* ATCC 25923 was purified using QIAquick PCR Purification Kit (Qiagen, Germany) and sequenced by 3730 × l DNA Analyzer (Applied Biosystems, USA). Similarity searches for the nucleotide sequences were performed with BLASTN program (http://www.ncbi.nlm.nih.gov/blast) using default settings.

#### Determination of *spd*C gene expression

The level of *spdC* gene expression was determined by quantitative real-time PCR (qRT-PCR). The previously described primers for *spd*C gene detection, in the “Detection of *spdC* gene” section, were used in qRT-PCR. 16S rRNA was used as a housekeeping gene; the sequences of the primers used for 16S rRNA amplification were as follows: *16S_Forward*: GTGGAGGGTCATTGGAAACT, and *16S_Reverse*: CACTGGTGTTCCTCCATATCTC.

Tested strains were allowed to grow in TSB with shaking at 180 rpm until the OD of the culture at 600 nm reached 1 (Poupel et al. 2018). RNA extraction was carried out using RNeasy mini kit (Qiagen, Germany) as per manufacturer’s protocol. The extracted RNA was used (400 ng) for reverse-transcription and amplification of the produced cDNA using iTaq™ Universal SYBR® Green One-Step Kit (Bio-Rad, USA) following manufacturer’s protocol. The amplification of cDNA was conducted with Rotor-Gene 6000 real-time thermal cycler (Corbett Life Science, Mortlake, Australia) according to the manufacturer’s instructions. The level of *spd*C gene expression in each tested isolate was normalized to the 16S rRNA gene expression level as a housekeeping gene and expressed as fold change relative to that in the reference *S. aureus* ATCC 25923 strain as a calibrator.

### Statistical analysis

Kendall’s Tau correlation coefficient was used to test the correlation between the fold change of *spd*C gene expression level and the activity of different virulence factors, as well as the resistance to different antibiotics. Kendall’s Tau correlation coefficient measures the strength of the relationship between two variables by assessing the statistical associations based on the ranks of data and is preferred for small sample size (Puka 2011). The Wilcoxon rank-sum test was used for the determination of the significance of the difference between the median of fold change in *spd*C gene expression level in isolates with different antimicrobial susceptibility phenotypes. The Wilcoxon rank-sum test is used for comparing the median of non-parametric data (Neuhäuser 2011).

## Results

**Fig. 1:**
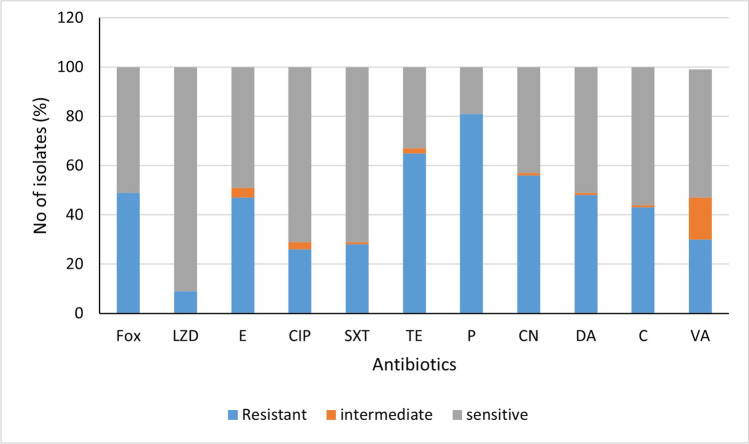
Antimicrobial susceptibility pattern of clinical *Staphylococcus aureus* isolates. Fox, cefoxitin; LZD, linezolid; E, erythromycin; CIP, ciprofloxacin; SXT, sulfamethoxazole-trimethoprim; TE, tetracycline; P, penicillin; CN, gentamicin; DA, clindamycin; C, chloramphenicol; VA, vancomycin

A total of 100 *S. aureus* clinical isolates were used in the study. Identification of isolates as *S. aureus* was confirmed by detection of *nuc* gene in all the collected isolates. Most of the isolates were MDR (81%) and nearly half of which were MRSA. Vancomycin resistance was detectable in 31% of the isolates. Most of *S. aureus* isolates were sensitive to linezolid (91%). Penicillin resistance was detectable in 81% of the tested isolates. Sensitivity to sulfamethoxazole/trimethoprim and ciprofloxacin was detected in 70% of the isolates (Fig. 1). Supplementary Table 1 describes the antimicrobial susceptibility pattern of each tested isolate.

### Virulence of *S. aureus* isolates

The activity of different virulence factors produced by the tested isolates was determined and the isolates were given arbitrary scores based on the activity of each virulence factor. The scores of each virulence factor activity in each isolate are given in Supplementary Table 3.

#### Delta-hemolysin activity

Delta-hemolysin activity was determined by measuring the diameter of hemolysis zone in sheep-blood agar plates. About 42% of the isolates had moderate hemolysin activity while 26% and 11% of the isolates had high and low hemolysin activity, respectively. The remaining isolates (21%) lacked any hemolysin activity under the same conditions. Representative results with different scores are given in Supplementary Fig. 1.

#### Extracellular protease activity

The extracellular protease activity was tested in skim milk agar plates. The protease activity was high in 17% of the isolates, moderate in 43% of the isolates, and weak in 11% of the isolates, while 29% of the isolates lacked any protease activity. Representative results with different scores are given in Supplementary Fig. 2.

#### Biofilm production

Out of 100 tested isolates, only 4% were non-biofilm producers including 6% of MRSA isolates, while 62% were weak biofilm producers including 57% of MRSA isolates. Moderate biofilm formation was detectable in 31% of the tested isolates (*n* = 31) including nearly 35% of MRSA isolates, while strong biofilm production was detectable in three isolates including 2% of the isolates that were MRSA. Representative example of biofilms formed by isolates with different scores is given in Supplementary Fig. 3.

**Fig. 2:**
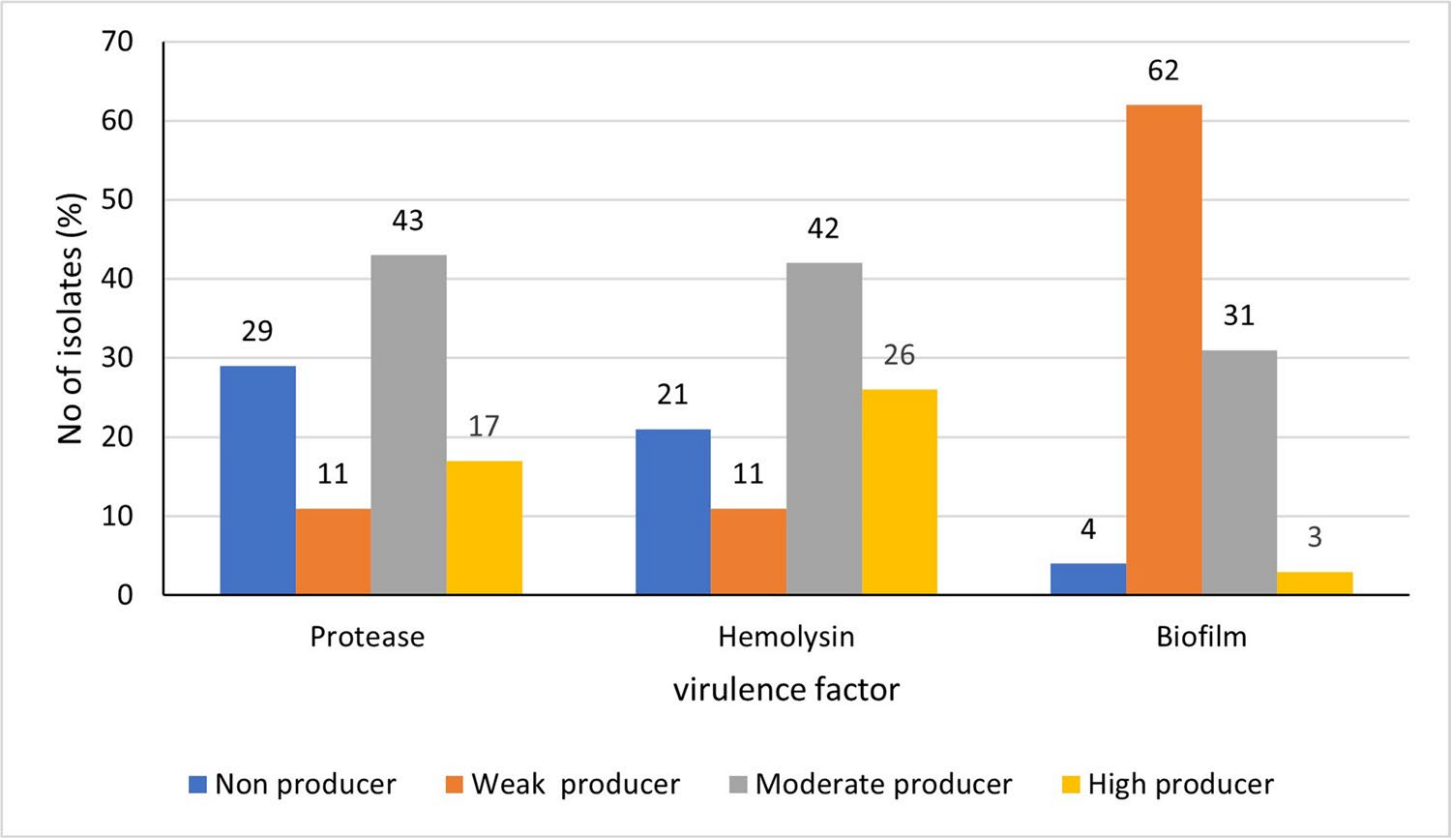
Production level of different virulence factors by the tested *Staphylococcus aureus* clinical isolates

The level for production of each virulence factor by tested *S. aureus* clinical isolates is given in Fig. 2. The total virulence score of each isolate was determined by sum up of the scores recorded for the three measured virulence determinants (protease, hemolysin, and biofilm) and ranged from 0 to 9 (Supplementary Table 3).

### Detection and expression level of *spd*C gene

#### Detection of *spd*C gene

*spd*C gene was detected in all the tested isolates by conventional PCR. Supplementary Fig. 4 shows representative examples of PCR products of *spd*C gene amplification visualized on agarose gel electrophoresis. Sequencing of the resulting PCR product from *S. aureus* ATCC 25923 confirmed 100% identity of the amplified product to the corresponding sequence of *spd*C gene of *S. aureus*. The sequenced product was deposited in GenBank under accession number ON367942.

#### Level of *spd*C gene expression

Due to financial limitations, the level of *spd*C gene expression was determined in eight selected isolates only. These isolates represented different virulence levels and antimicrobial resistance (AMR) patterns as follows: MRSA and high virulence score (isolates 13 and 78, total score = 8), MRSA and low virulence score (isolates 33 and 83, total score = 1 and 2, respectively), MSSA and high virulence score (isolates 65 and 25, total score = 8 and 7, respectively), and MSSA and low virulence score (isolates 93 and 30, total score = 0 and 1, respectively).

The specificity of the used primers was confirmed by the melting curves of the amplification products that showed only one single peak (Supplementary Fig. 5). *spd*C expression level varied between 0.08 and 1.18 that recorded in the standard *S. aureus* ATCC 25923. Five isolates had *spd*C expression level < 0.6 that of the standard strain. Three of these isolates were MRSA including two with a high virulence score (isolates 78 and 13) and one with a low virulence score (isolate 33). The other two isolates were MSSA and had a high virulence score (isolates 25 and 65). Three isolates had *spd*C expression level > 0.6 that of the standard *S. aureus* ATCC 25923; all had a low virulence score: one was MRSA (isolate 83) and the other two were MSSA (isolates 30 and 93; Table 1).

**Table 1:** Virulence factors’ arbitrary scores in tested isolates and their calculated Kendall’s Tau correlation coefficient with *spd*C gene expression level

Isolate number	Virulence factor score				Fold change in *spd*C expression level relative to that in *S. aureus* ATCC 25923
	Protease	Hemolysin	Biofilm	Total	
13	2	3	3	8	0.59778
25	2	2	3	7	0.08153
30	0	0	1	1	1.18237
33	0	0	1	1	0.2881
65	3	3	2	8	0.16916
78	3	3	2	8	0.32279
83	1	1	0	2	0.65784
93	0	0	0	0	0.73267
*S. aureus* ATCC 25923	2	2	3	7	1
Kendall’s Tau correlation coefficient	− 0.4	− 0.34	− 0.24	− 0.33	
*p* value	0.16	0.23	0.39	0.24	

#### Correlation between *spd*C gene expression level and virulence

Discordant moderate correlation was recorded between the level of expression of *spdC* gene and the activity of the tested virulence factors, using Kendall’s Tau correlation coefficient. However, this correlation was not significant (*p* = 0.16, 0.23, 0.39, or 0.24 for protease, hemolysin, biofilm, or the total virulence scores, respectively; Table 1).

#### Correlation between *spd*C gene expression level and AMR

Moderate concordant correlation was recorded between *spd*C gene expression level and the AMR to vancomycin, linezolid, tetracycline, gentamicin, clindamycin, and chloramphenicol, while weak concordant correlation was recorded with erythromycin resistance, using Kendall’s Tau correlation coefficient. On the other hand, Kendall’s Tau correlation coefficient indicated discordant correlation between *spd*C gene expression level and the AMR in the case of sulfamethoxazole/trimethoprim (weak correlation) and ciprofloxacin (moderate correlation). This correlation between *spd*C gene expression level and the AMR whether concordant or discordant, measured using Kendall’s Tau correlation coefficient, was insignificant (*p* > 0.05).

The median of *spd*C gene expression level was higher in resistant isolates, compared to susceptible isolates, to most of the tested antibiotics (vancomycin, linezolid, erythromycin, tetracycline, gentamicin, clindamycin, and chloramphenicol). In the case of sulfamethoxazole/trimethoprim and ciprofloxacin, lower median of *spd*C gene expression level was detected in resistant isolates. However, in all tested antibiotics, no significant difference was detectable between the median of *spd*C gene expression level in isolates with different resistance pattern, using the Wilcoxon rank-sum test (Supplementary Table 4).

Weak discordant correlation was recorded between methicillin resistance (cefoxitin resistance) phenotype and the *spd*C gene expression level, with higher median of *spd*C gene expression in MSSA isolates. However, no significant correlation was recorded between *spd*C gene expression level and MRSA phenotype using either Kendall’s Tau correlation coefficient (*p *= 0.62) or the Wilcoxon rank-sum test (*p* = 0.73).

## Discussion

*S. aureus* expresses a vast array of virulence factors that impact disease progression and severity (Liu 2009). Antibiotic resistance and emergence of MDR isolates, in addition to unavailability of effective vaccine, complicate the treatment of *S. aureus* infections (Cheung et al. 2021). SpdC protein was described as a global regulator of *S. aureus* virulence that affects biofilm formation and pathogenesis in addition to altering resistance toward compounds targeting cell wall (Poupel et al. 2018).

The rates of antimicrobial susceptibility patterns and virulence in our isolates were similar to those recorded elsewhere. AMR was predominant among the tested isolates, where 81% of the isolates were MDR and about half of the isolates (49%) were MRSA. Similar rates of MRSA infection were reported previously in Egypt, Africa, and the Middle East (Elshimy et al. 2018; Elsayed et al. 2018; Zigmond et al. 2014), as well as in other regions worldwide (Diekema et al. 2019).

High rates of resistance to tested antibiotics such as penicillin, cefoxitin, tetracycline, clindamycin, erythromycin, gentamicin, and chloramphenicol were also detectable. Most of the isolates (81%) were penicillin-resistant; this penicillin resistance rate is slightly lower than those recorded in other studies in 2018 and 2020, where penicillin resistance rates exceeded 90% (Elsayed et al. 2018; Kashef et al. 2020; Manandhar et al. 2018). Similar observations regarding the increased susceptibility to penicillin in *S. aureus* were described previously (Butler-Laporte et al. 2018; Chabot et al. 2015). This might arise from the limited use of penicillin in treatment of *S. aureus* infections where restricting the use of a certain antimicrobial may help in restoring its activity (Baym et al. 2016; Maher et al. 2012).

Vancomycin and linezolid are considered among the last-line treatments of MRSA infections. Resistance to linezolid was detected in 9% of the tested isolates which was higher than the rates recorded previously for linezolid resistance in Egypt (Kashef et al. 2020) and other regions of the world (Gu et al. 2013; Quiles-Melero et al. 2013). Vancomycin resistance was detected in 31% of the tested isolates. This is a considerably high rate, where vancomycin is the drug of choice for MRSA treatment. Higher and lower rates of vancomycin resistance were recorded previously in Egypt (Al-Amery et al. 2019; Elsayed et al. 2018; Kashef et al. 2020; El Refai et al. 2014) and other areas (Alzolibani et al. 2012; Yilmaz and Aslantaş 2017). According to the meta-analysis carried by Wu et al. (2021), the highest prevalence of vancomycin-resistant *S. aureus* was reported in Africa (16%) with Nigeria having the highest prevalence rate (29%). In the USA, the threat of vancomycin resistance was reduced (CDC 2019).

Biofilm formation by *S. aureus* plays an important role in chronic disease progression and increases tolerance to antibiotics (Lister and Horswill 2014). All except four of the collected isolates were biofilm-forming (96%) which was similarly reported previously (Kashef et al. 2020; Omidi et al. 2020; Piechota et al. 2018). In addition, about 95% of the MDR isolates were biofilm-forming. The high rates of biofilm formation among MDR isolates were recorded in previous studies (Kwon et al. 2008). Similarly, we detected moderate number of isolates with the ability to produce other tested virulence factors as protease and hemolysin (71% and 79%, respectively).

All tested isolates carried *spd*C gene in their genome. This is in accordance with a previous study that confirmed the presence of *spd*C gene in all sequenced staphylococci isolates (Gründling et al. 2006). However, Yamaguchi et al. (2019) recently detected a vancomycin intermediate–resistant *S. aureus* strain that lacked *spd*C gene. In our study, we isolated 17 strains with intermediate vancomycin resistance and none of our strains lacked the *spd*C gene. Other mechanisms might be responsible for vancomycin intermediate resistance phenotype as single nucleotide polymorphisms in genes responsible for cell wall biosynthesis. The effect of *spd*C gene deletion on vancomycin resistance needs to be further confirmed.

Interestingly, there was no significant correlation between biofilm formation and the level of the *spd*C gene expression in our clinical isolates. The measured Kendall’s Tau correlation coefficient between *spd*C gene expression level and biofilm formation indicated moderate discordant correlation. This opposed the results reported earlier about highly reduced biofilm formation in *spd*C mutant (Poupel et al. 2018). No studies are available on the correlation between biofilm formation and *spd*C gene expression in clinical isolates.

This may indicate the indirect effect of SpdC on biofilm formation. Other regulators are documented to be implicated directly in controlling biofilm formation as WalKR, RNAIII, SarA, and SigB. SpdC negatively affects the expression of WalKR regulon which in turn is known to positively affect biofilm formation (Dubrac et al. 2007; Paharik and Horswill 2016; Poupel et al. 2018). Other studies are still required to elucidate the exact molecular mechanism for SpdC effect on biofilm formation.

Similar to the lack of significant correlation between *spd*C gene expression level and biofilm formation, we also failed to find any significant correlation between *spd*C gene expression level and extracellular protease activity in our isolates. Proteases are important virulence factors that can cleave and degrade several important host proteins, including the heavy chains of all human immunoglobulin classes, plasma proteinase inhibitor, and elastin. In addition, proteases also play a role in *S. aureus* invasiveness by degrading bacterial cell surface proteins responsible for bacterial adhesion such as protein A and fibronectin-binding protein (Karlsson and Arvidson 2002).

*S. aureus* produces a number of proteases. These include two cysteine proteases (staphopain A, ScpA; and staphopain B, SspB), a metalloprotease (aureolysin), a serine protease (V8 or SspA), and six serine-like proteases (Spls) that are SspA homologs (*Spl*ABCDEF) (Lehman et al. 2019). Synthesis of extracellular proteases is activated by the accessory gene regulator quorum-sensing system (Agr) and repressed by SarA protein (Lehman et al. 2019). SpdC was suggested to activate SplB and SplC (serine-like proteases B and C) and staphopain thiol protease (SspB) (Poupel et al. 2018). We reported discordant correlation between *spd*C gene expression level and the phenotypic protease activity. This may be caused by the predominant effect of one or more proteases over the others and/or the predominant effect of other regulators on proteases’ expression.

Also, no significant correlation between *spd*C expression level and delta-hemolysin activity was detectable; however, moderate discordant association was recorded. Poupel et al. (2018) reported the lack of SpdC effect on *S. aureus* delta-hemolysin gene. SpdC induces the expression of gamma-hemolysin; this may be due to the positive effect of SpdC on the expression of the TCS *sae*RS and its negative effect on the WalKR system (Poupel et al. 2018). However, delta-hemolysin production in *S. aureus* is mainly regulated by the Agr system (Divyakolu et al. 2019).

Similar to the results of Poupel et al. (2018) about the increased susceptibility to cell wall active agents in *spd*C mutant, we found a moderate concordant correlation between *spd*C gene expression level and the AMR especially in cell wall active agents as vancomycin and penicillin. However, this correlation was not significant. These agents are cell wall antibiotics that inhibit the late stages of cell wall biosynthesis (Sarkar et al. 2017). Only with methicillin (cefoxitin) resistance, discordant correlation was recorded.

The effect of SpdC on the susceptibility to antibiotics with targets other than cell wall biosynthesis was also tested in this study (ciprofloxacin, sulfamethoxazole/trimethoprim, chloramphenicol, erythromycin, clindamycin, tetracycline, gentamicin, and linezolid), where no significant correlation was detectable between their susceptibility pattern and *spd*C gene expression level. Although, there was moderate concordant correlation between *spd*C gene expression level and the AMR to these agents, except with ciprofloxacin and sulfamethoxazole/trimethoprim, where discordant correlation was detectable.

Collectively, we failed to record any significant correlation between *spd*C gene expression level and virulence or antimicrobial susceptibility in clinical *S. aureus* isolates. Similar observation regarding the lack of direct effect of *spd*C and other *spd* genes on gene transcription or translation was reported previously (Frankel et al. 2010). This may reflect the predominant effect of other virulence regulators in *S. aureus* as WalKR, Agr, SaeRS, SrrAB, ArlSR, and LytRS, in addition to *Sar*A and *Sar*A homologs (Pragman and Schlievert 2004; Bronner et al. 2003). However, these regulators might be partially affected by SpdC level indicated by the moderate correlation detected in most cases.

Previous studies have reported the predominant effect of one regulator over the other on various virulence factors such as the predominant negative effect of SarA over the positive effect of the Agr system on the protease activity (Karlsson and Arvidson 2002), also the dominant effect of Sae regulator over the effect of σ^B^ regulator on virulence gene expression in *S. aureus* during device-related infection (Goerke et al. 2005). Single nucleotide polymorphism in *spd*C gene might affect the level of different virulence regulators that are affected by SpdC.

The results of this study are preliminary, and more studies are still required to confirm this conclusion. This study has a limitation of the small number of isolates tested for *spd*C gene expression level (eight isolates). Testing this association on larger number of isolates is urgently required to confirm the results of this study together with the possible effect of the isolate source. *spd*C mutants need to be prepared from clinical isolates to confirm the role of SpdC on virulence and antimicrobial resistance. Also, the association between *spd*C gene expression level and the level of expression of other virulence genes needs to be tested. In addition, the exact mechanism of *spd*C effect on the expression and regulation of different virulence factors is required to be elucidated.

## Conclusion

The increased rates of antibiotic resistance together with the prevalence of virulent strains in hospitals is alarming and urges for an effective infection control strategy. The previously reported role of SpdC protein as virulence regulator in *S. aureus* isolates needs further evaluation together with the determination of the predominant regulators for each virulence factor.

## Supplementary Information

Below is the link to the electronic supplementary material.

Supplementary file1 (XLSX 22 KB)

Supplementary file2 (DOCX 27 KB)

Supplementary file3 (XLSX 14 KB)

Supplementary file4 (PDF 94 KB)

Supplementary file5 (PDF 96 KB)

Supplementary file6 (PDF 215 KB)

Supplementary file7 (PDF 141 KB)

Supplementary file8 (PDF 284 KB)

Supplementary file9 (PDF 76 KB)
