# Supplementary material for: Effect of spdC gene expression on virulence and antibiotic resistance in clinical Staphylococcus aureus isolates
Source: Int Microbiol. 2022 May 24;25(3):649–59. doi: 10.1007/s10123-022-00249-6 (PMC9307553; doi:10.1007/s10123-022-00249-6)
Supplement: Supplementary file 2 — Supplementary file2 (DOCX 27 KB) [file 10123_2022_249_MOESM2_ESM.docx]

**Supplementary Table 2:** Zone Diameter Breakpoints used for determination of antimicrobial susceptibility according to CLSI (2017) guidelines

| **Antibiotic name (code)** | **Disc content (µg)** | **Interpretive criteria (inhibition zone diameter, mm)** | | |
| --- | --- | --- | --- | --- |
|  |  | **S** | **I** | **R** |
| Cefoxitin (Fox) | 30 | ≥22 | - | ≤21 |
| Chloramphenicol (C) | 30 | ≥18 | 13-17 | ≤12 |
| Ciprofloxacin (CIP) | 5 | ≥21 | 16-20 | ≤15 |
| Clindamycin (DA) | 2 | ≥21 | 15-20 | ≤14 |
| Erythromycin (E) | 15 | ≥23 | 14-22 | ≤13 |
| Gentamicin (CN) | 10 | ≥15 | 13-14 | ≤12 |
| Linezolid (LZD) | 30 | ≥21 | - | ≤20 |
| Penicillin (P) | 10 | ≥ 29 | - | ≤28 |
| Trimethoprim/ Sulfamethoxazole (SXT) | 1.25/23.75 | ≥16 | 11-15 | ≤10 |
| Tetracycline (TE) | 30 | ≥19 | 15-18 | ≤14 |
