# Supplementary material for: Effect of spdC gene expression on virulence and antibiotic resistance in clinical Staphylococcus aureus isolates
Source: Int Microbiol. 2022 May 24;25(3):649–59. doi: 10.1007/s10123-022-00249-6 (PMC9307553; doi:10.1007/s10123-022-00249-6)
Supplement: Supplementary file 4 — Supplementary file4 (PDF 94 KB) [file 10123_2022_249_MOESM4_ESM.pdf]

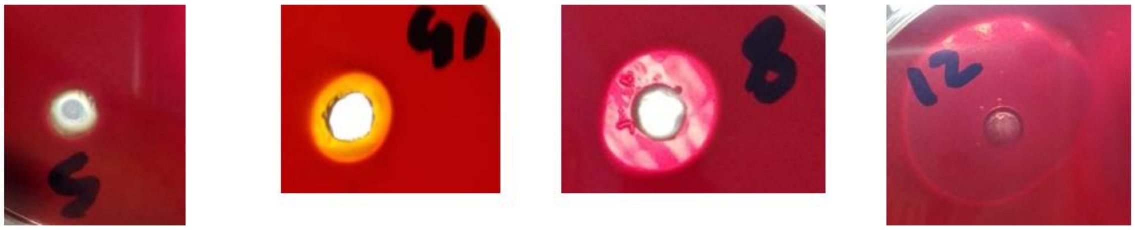

Isolate 5 (hemolysis score = 0) | Isolate 41 (hemolysis score = 1) | Isolate 8 (hemolysis score = 2) | Isolate 12 (hemolysis score = 3)

Supplementary Fig. 1 Representative results of hemolysis zone in sheep-blood agar plates from isolates with different hemolysis scores
