# Supplementary material for: Effect of spdC gene expression on virulence and antibiotic resistance in clinical Staphylococcus aureus isolates
Source: Int Microbiol. 2022 May 24;25(3):649–59. doi: 10.1007/s10123-022-00249-6 (PMC9307553; doi:10.1007/s10123-022-00249-6)
Supplement: Supplementary file 5 — Supplementary file5 (PDF 96 KB) [file 10123_2022_249_MOESM5_ESM.pdf]

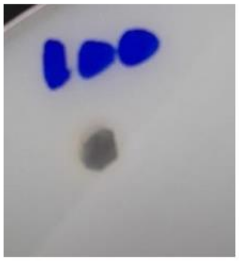

Isolate 100 (proteolysis score = 0)

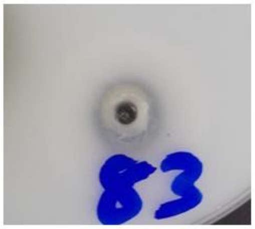

Isolate 83 (proteolysis score = 1)

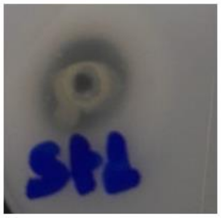

Standard strain (proteolysis score = 2)

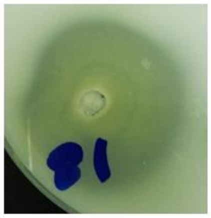

Isolate 81 (proteolysis score = 3)

Supplementary Fig. 2 Representative results of proteolysis zone in skim milk agar plates from isolates with different proteolysis scores
