# Supplementary material for: Effect of spdC gene expression on virulence and antibiotic resistance in clinical Staphylococcus aureus isolates
Source: Int Microbiol. 2022 May 24;25(3):649–59. doi: 10.1007/s10123-022-00249-6 (PMC9307553; doi:10.1007/s10123-022-00249-6)
Supplement: Supplementary file 6 — Supplementary file6 (PDF 215 KB) [file 10123_2022_249_MOESM6_ESM.pdf]

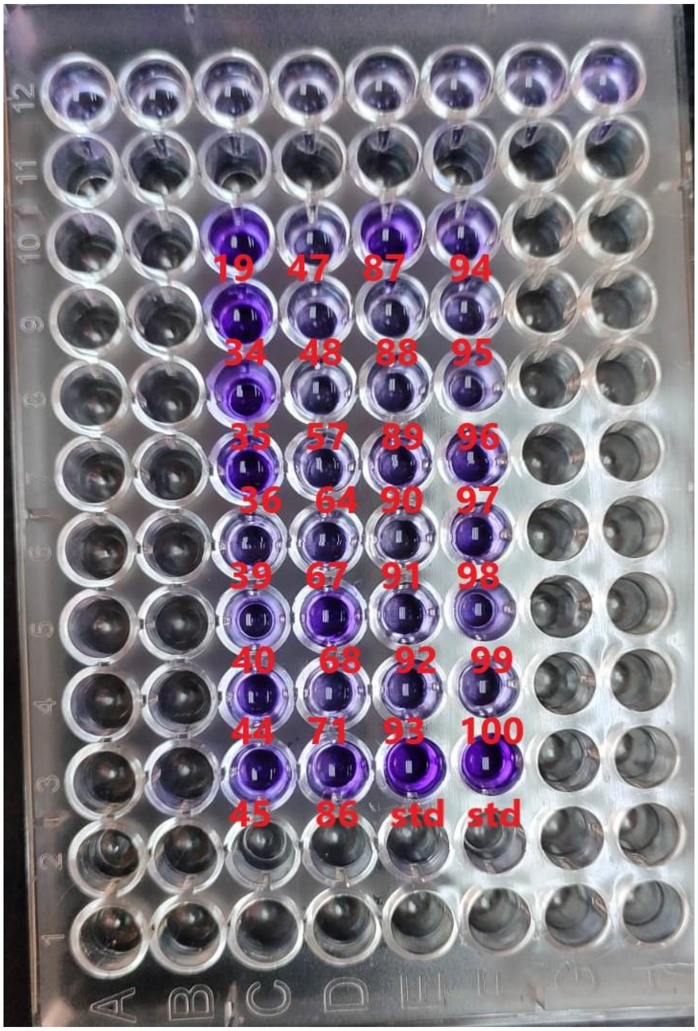

Supplementary Fig. 3 Representative results of biofilm formation from isolates with different biofilm scores
