# Supplementary material for: Effect of spdC gene expression on virulence and antibiotic resistance in clinical Staphylococcus aureus isolates
Source: Int Microbiol. 2022 May 24;25(3):649–59. doi: 10.1007/s10123-022-00249-6 (PMC9307553; doi:10.1007/s10123-022-00249-6)
Supplement: Supplementary file 7 — Supplementary file7 (PDF 141 KB) [file 10123_2022_249_MOESM7_ESM.pdf]

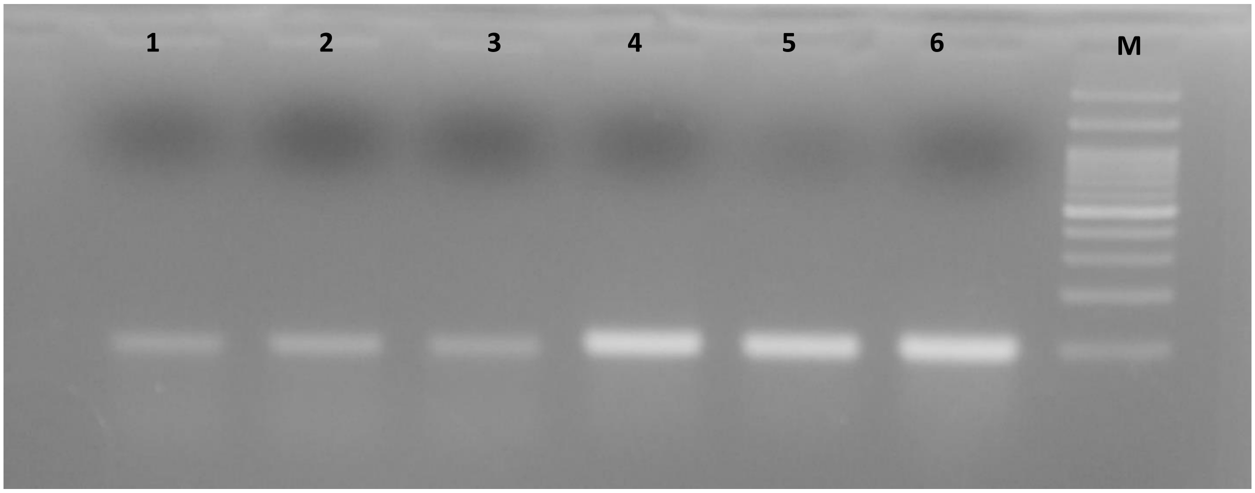

Supplementary Fig. 4 Amplified PCR products visualized on agarose gel; **Lanes 1 to 3:** *spd*C gene products of S. aureus ATCC 25923, isolate 78 and isolate 13, respectively; **Lanes 4 to 6:** *16S rRNA* products of S. aureus ATCC 25923, isolate 78 and isolate 13, respectively; **M:** 100 bp ladder
