# Supplementary material for: Effect of spdC gene expression on virulence and antibiotic resistance in clinical Staphylococcus aureus isolates
Source: Int Microbiol. 2022 May 24;25(3):649–59. doi: 10.1007/s10123-022-00249-6 (PMC9307553; doi:10.1007/s10123-022-00249-6)
Supplement: Supplementary file 8 — Supplementary file8 (PDF 284 KB) [file 10123_2022_249_MOESM8_ESM.pdf]

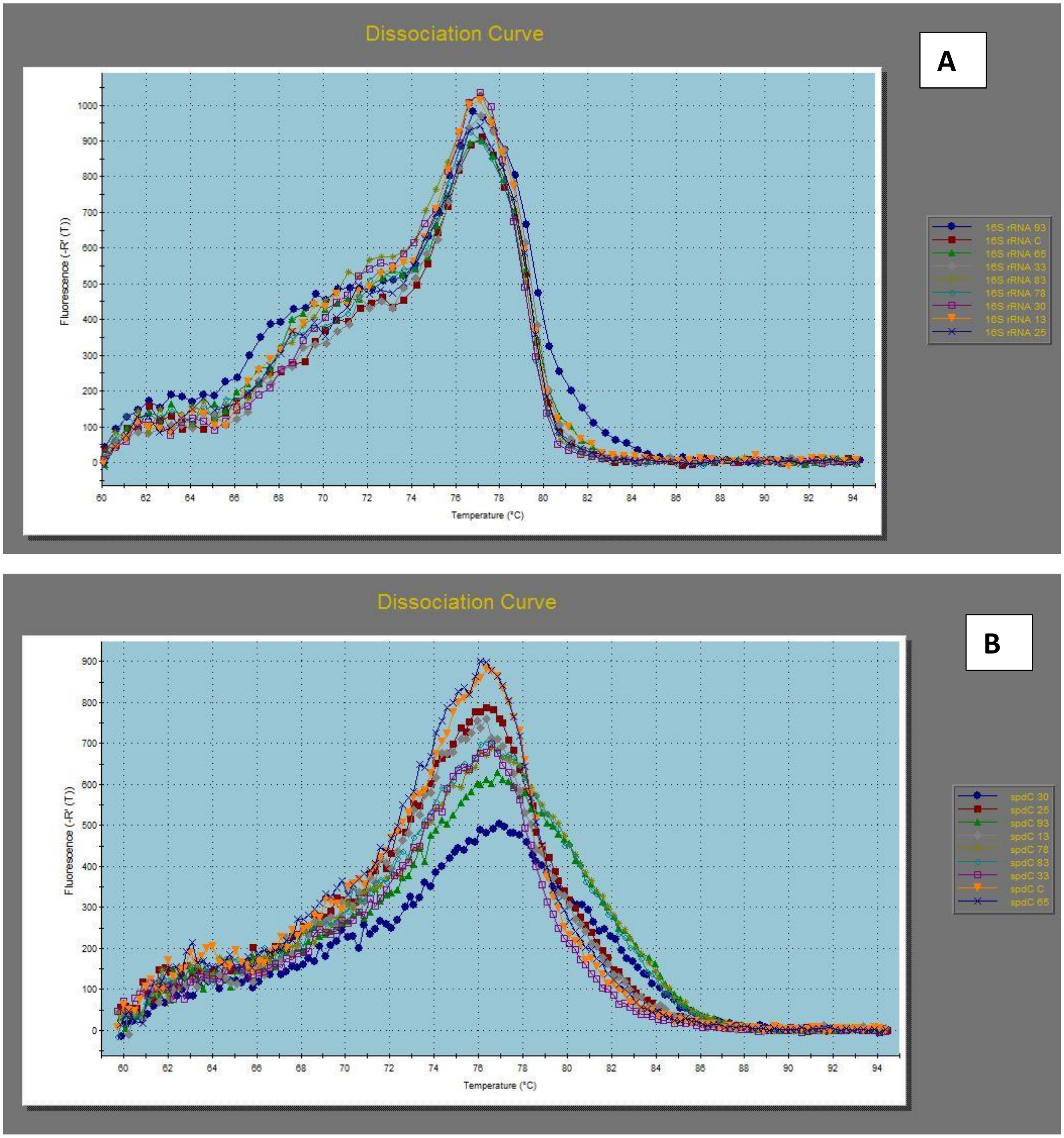

Supplementary Fig. 5 Melting curves of amplification products of qRT-PCR performed using **A:** *16S* rRNA primers and **B:** *spd*C primers
