# Supplementary material for: Effect of spdC gene expression on virulence and antibiotic resistance in clinical Staphylococcus aureus isolates
Source: Int Microbiol. 2022 May 24;25(3):649–59. doi: 10.1007/s10123-022-00249-6 (PMC9307553; doi:10.1007/s10123-022-00249-6)
Supplement: Supplementary file 9 — Supplementary file9 (PDF 76 KB) [file 10123_2022_249_MOESM9_ESM.pdf]

Supplementary Table 4 Statistical correlation between *spd*C gene expression level and the susceptibility to different antibiotics

**Antimicrobial susceptibility pattern**	**Kendall's Tau correlation coefficient**	***p* value (Kendall's Tau correlation coefficient)**	**Median of *spd*C fold-change relative to *S. aureus* ATCC 25923**	***p* value (Wilcoxon rank sum test)**
**MRSA[1] (cefoxitin resistant)**	-0.15	0.62	0.46	0.73
**MSSA[2] (cefoxitin susceptible)**			0.733	
**Vancomycin resistant**	0.39	0.2	0.733	0.262
**Vancomycin sensitive**			0.443	
**Linezolid resistant**	0.24	0.44	0.733	0.667
**Linezolid sensitive**			0.46	
**Erythromycin resistant**	0.07	0.81	0.644	0.905
**Erythromycin sensitive**			0.598	
**Ciprofloxacin resistant**	-0.47	0.12	0.0815	0.222
**Ciprofloxacin sensitive**			0.628	
**Sulfamethoxazole/trimethoprim resistant**	-0.06	0.82	0.202	0.6
**Sulfamethoxazole/trimethoprim sensitive**			0.473	
**Sulfamethoxazole/trimethoprim intermediate**			1	
**Tetracycline resistant**	0.15	0.61	0.665	1
**Tetracycline sensitive**			0.49	
**Tetracycline intermediate**			0.169	
**Penicillin resistant**	0.00	1.00	0.598	1
**Penicillin sensitive**			0.585	
**Gentamicin resistant**	0.3	0.33	0.829	0.413
**Gentamicin sensitive**			0.323	
**Clindamycin resistant**	0.22	0.46	0.733	0.556
**Clindamycin sensitive**			0.46	
**Chloramphenicol resistant**	0.52	0.09	0.733	0.111
**Chloramphenicol sensitive**			0.246	

[1]**MRSA:** Methicillin resistant *Staphylococcus aureus*, [2]**MSSA:** Methicillin susceptible *Staphylococcus aureus*
